# Diagnostic Application of Targeted Resequencing for Familial Nonsyndromic Hearing Loss

**DOI:** 10.1371/journal.pone.0068692

**Published:** 2013-08-22

**Authors:** Byung Yoon Choi, Gibeom Park, Jungsoo Gim, Ah Reum Kim, Bong-Jik Kim, Hyo-Sang Kim, Joo Hyun Park, Taesung Park, Seung-Ha Oh, Kyu-Hee Han, Woong-Yang Park

**Affiliations:** 1 Department of Otorhinolaryngology, Seoul National University Bundang Hospital, Seongnam, Korea; 2 Department of Biomedical Sciences, College of Medicine, College of Natural Science, Seoul National University, Seoul, Korea; 3 Interdiciplinary Program for Bioinformatics, College of Natural Science, Seoul National University, Seoul, Korea; 4 Statistics, College of Natural Science, Seoul National University, Seoul, Korea; 5 Otorhinolaryngology, Seoul National University Hospital, Seoul, Korea; 6 Department of Molecular and Cellular Biology, Sungkyunkwan University School of Medicine, Suwon, Korea; 7 Samsung Genome Institute, Samsung Medical Center, Seoul, Korea; Ajou University, Korea, Republic of

## Abstract

Identification of causative genes for hereditary nonsyndromic hearing loss (NSHL) is important to decide treatment modalities and to counsel the patients. Due to the genetic heterogeneity in sensorineural genetic disorders, the high-throughput method can be adapted for the efficient diagnosis. To this end, we designed a new diagnostic pipeline to screen all the reported candidate genes for NSHL. For validation of the diagnostic pipeline, we focused upon familial NSHL cases that are most likely to be genetic, rather than to be infectious or environmental. Among the 32 familial NSHL cases, we were able to make a molecular genetic diagnosis from 12 probands (37.5%) in the first stage by their clinical features, characteristic inheritance pattern and further candidate gene sequencing of *GJB2*, *SLC26A4*, *POU3F4* or mitochondrial DNA. Next we applied targeted resequencing on 80 NSHL genes in the remaining 20 probands. Each proband carried 4.8 variants that were not synonymous and had the occurring frequency of less than three among the 20 probands. These variants were then filtered out with the inheritance pattern of the family, allele frequency in normal hearing 80 control subjects, clinical features. Finally NSHL-causing candidate mutations were identified in 13(65%) of the 20 probands of multiplex families, bringing the total solve rate (or detection rate) in our familial cases to be 78.1% (25/32) Damaging mutations discovered by the targeted resequencing were distributed in nine genes such as *WFS1*, *COCH*, *EYA4*, *MYO6*, *GJB3*, *COL11A2*, *OTOF*, *STRC* and *MYO3A*, most of which were private. Despite the advent of whole genome and whole exome sequencing, we propose targeted resequencing and filtering strategy as a screening and diagnostic tool at least for familial NSHL to find mutations based upon its efficacy and cost-effectiveness.

## Introduction

Hearing loss is a common sensorineural disorder affecting one out of 500 live births, with increasing prevalence into adolescence [1]. While there are many environmental causes of hearing loss, such as viral infections, acoustic trauma, and ototoxic drugs, approximately half of all cases are hereditary [2]. Genetic causes of hearing loss can be detected by sequence analysis, which helps clinicians and patients to delineate the characteristics of disease. In addition, hearing loss occurring in early childhood can affect the linguistic development [3], so it is quite important to improve our techniques to find genetic alterations in patients for further clinical care of this disease.

Nonsyndromic hearing loss (NSHL) contribute 70% of inherited hearing loss, and most of NSHL were autosomal recessive up to 80%, in comparison to 20% autosomal dominant and less than 1% X-linked or mitochondrial disorders [4]. About 46 genes have been identified and causally related to nonsyndromic hearing loss. However, over 100 loci have been mapped for monogenic hearing loss, with specific genes yet to be pinpointed [1]. The sheer complexity of the auditory system accounts for the large number of genes and loci linked to hearing loss.

Studies in hearing loss genes have increased rapidly with the advent of next-generation sequencing (NGS). NGS allows whole genome sequencing to be done quickly at a much lower cost than Sanger sequencing. Whole genome, whole exome, and targeted gene sequencing has become far more feasible, allowing for easier identification of disease genes [5]. Identification of deafness genes has several clinical implications. Because hearing loss is oftentimes monogenic, genetic testing can accurately predict the deafness phenotype. Genetic testing using NGS could provide an accurate, definitive answer with eliminating the need for further expensive testing [5]. Identification of genes and genetic testing will also allow the specific cause of a patient's hearing loss to be uncovered, which cannot be identified by Universal Newborn Hearing Screening [5]. Knowing the cause of hearing loss will allow prediction of the efficacy of certain therapeutic approaches, such as cochlear implantation, and in the future might allow for the development of further therapeutics and protective medications [3].

Here we report the new diagnostic pipeline combining Sanger sequencing and targeted resequencing to find mutations in familial NSHL cases. Screening mutations in all exons in 80 reported deafness genes could detect candidate mutations in 13 (65%) out of 20 familial NSHL cases. Together with Sanger sequencing against four NSHL genes, the mutation detection rate was increased to 78.1% (25/32).

## Materials and Methods

### Ethics statement

This study was approved by the Institutional Review Boards (IRBs) at Seoul National University Bundang Hospital (IRB-B-1007-105-402 and IRB-B-1111-139-015) and Seoul National University Hospital (IRBY-H-0905-041-281). We obtained a written informed consent from all the participants in this study. In case of children participants, the written informed consent was obtained from the parents or guardians on behalf of them.

### Patient selection

Among 145 hearing impaired probands who visited our tertiary referral center and who were willing to participate the genetic test from May 2010 through April 2012, 30 probands with syndromic features were excluded. Among the remaining 115 probands, 31 families with at least two or more hearing-impaired members without any syndromic feature (multiplex families) were selected and blood samples were taken. Medical histories were collected including age at onset of hearing loss, degree and progression of hearing impairment, and other relevant clinical manifestations.

### DNA preparation, Sanger sequencing and targeted resequencing

Genomic DNAs were extracted from peripheral blood as described previously [6]. Sanger sequencing was performed using specific primers for each exon as described (Table S1). Targeted exome sequencing was done by Otogenetics (Norcross, GA). Briefly, genomic DNA was used for NimbleGen capture methods (Roche NimbleGen Inc., Madison, WI) against 80 known deafness genes (Table S1). An additional 50 bp of flanking intronic sequence were added to each exon and genomic intervals were merged using Galaxy software (http://galaxy.psu.edu). In total, we targeted 1,258 regions comprising 421,741 bp using NimbleGen methods.

### Alignment, coverage calculation and variant detection

Reads were aligned to UCSC hg19 reference genome using BWA-0.6.1 with default settings [6]. To process sam/bam files and mark duplicates, Samtools and Picards were used. Local realignment around indels and base quality score recalibration were done for each samples and variants were called by unified genotyper in GATK-1.3. Perl script and Annovar were used to annotate variants and search the known SNPs and indels from dbsnp135 and 1000 genome draft. Coverages were calculated by GATK.

### Model of independent uncaptured exons

To evaluate the correlation of capture performance between each samples, we compared experimental and expected distribution in number of exons that were commonly uncaptured within from 0 to 20 samples. Expectation number was calculated by the model assuming that uncapturing of exons occur independently between samples. Binomial model was not used because the difference of the numbers of uncaptured exons was not ignorable. We defined that uncaptured exon is an exon within which % of bases above 10, 50 or 100 of read depth is less than 1%.

p_k_: ratio of uncaptured exons in k^th^ sample

P(# = n): probability that number of samples having uncaptured exons in common is n

Here, the number of samples is 20 and the number of exons is 1254.
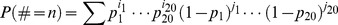
(sum for all combinations where, 
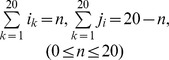
)

Due to too large number of combinations, each probability(P) was calculated with permutation 1,000 times with Python using a module “decimal” for precision, instead of summing all the combinations (but P(# = 0) was calculated directly.). Then, expected counts were obtained.
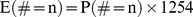



## Results

We have collected 145 sensorineural hearing loss cases for a molecular genetic diagnosis in SNUH and SNUBH. Especially 32 multiplex familial cases were focused to find genetic aberrations for diagnosis and genetic counseling because we can validate the causative mutation through co-segregation in the family. We established the new diagnostic pipeline combining PCR and targeted resequencing. Eleven cases showed either clearly defined phenotype related to the mutations in *SLC26A4*, *POU3F4* and mitochondrial DNA genes (Table 1 and Fig. S1). Temporal bone CT was taken to rule out any abnormality of the inner ear. Cases with characteristic radiologic markers such as bilateral enlarged vestibular aqueduct (5 probands) or incomplete partition type III (5 probands) were directly subject to further Sanger sequencing of the corresponding candidate genes, *SLC26A4* and *POU3F4*, respectively. Mitochondrial DNA was sequenced for one family that showed characteristic maternal inheritance of hearing loss. In these eleven families, we could successfully find mutations by PCR sequencing, which were mostly located in the reported sites (Table 1). *GJB2* sequencing was performed for the remaining 21 hearing impaired probands because the mutation in *GJB2* was most frequent among familial NSHL cases. We found two cases (SJ19-19 and SH35-75) with known pathogenic mutations in *GJB2* gene.

**Table 1:** Mutations of SLC26A4, POU3F4, GJB2 and MTRNR1 in 12 familial NSHL found by PCR-Sanger sequencing.

Patient	Characteristic phenotype	Gene	Mutation type	GeneBank No.	Chr	Exon	Nucleotide	Protein	MAF*	dbSNP135
SB02-6	Incomplete partition type III	POU3F4	Nonsynonymous	NM_000307	X	exon 1	c.686A>G	p.Gln229Arg	-	-
SB07-18	Incomplete partition type III	POU3F4	Frameshift deletion	NM_000307	X	exon 1	c.1060delA	p.Thr354GlnfsX115	-	-
SB08-19	Incomplete partition type III	POU3F4	Frameshift insertion	NM_000307	X	exon 1	c.950dupT	p.Leu317PhefsX12	-	-
SB09-21	Incomplete partition type III	POU3F4	Nonsynonymous	NM_000307	X	exon 1	c.632C>T	p.Thr211Met	-	-
SB13-29	Incomplete partition type III	POU3F4	stopgain	NM_000307	X	exon 1	c.623T>A	p.Leu208X	-	-
SB16-34	Nonsyndromic EVA	SLC26A4	Nonsynonymous	NM_000441	7	exon19	c.A2168G	p.H723R	0.001	rs121908362
SB23-54	Nonsyndromic EVA	SLC26A4	Nonsynonymous	NM_000441	7	exon19	c.A2168G	p.H723R	0.001	rs121908362
SB28-61	Nonsyndromic EVA	SLC26A4	Nonsynonymous	NM_000441	7	exon19	c.A2168G	p.H723R	0.001	rs121908362
SJ07-7	Nonsyndromic EVA	SLC26A4	Nonsynonymous	NM_000441	7	exon19	c.A2168G	p.H723R	0.001	rs121908362
SJ20-20	Nonsyndromic EVA	SLC26A4	Nonsynonymous	NM_000441	7	exon19	c.A2168G	p.H723R	0.001	rs121908362
SH07-19	Maternal transmission	MTRNR1	Nonsynonymous		Mt		1,555A>G		-	-
SJ19-19	no specific phenotype	GJB2	Frameshift deletion	NM_004004	13	exon2	c.299_300del	p.H100RfsX14	-	-
			Frameshift deletion	NM_004004	13	exon2	c.235delC	p.L79CfsX3	-	-

* MAF: minor allele frequency from 1,000 Genome.

**Figure 1:**
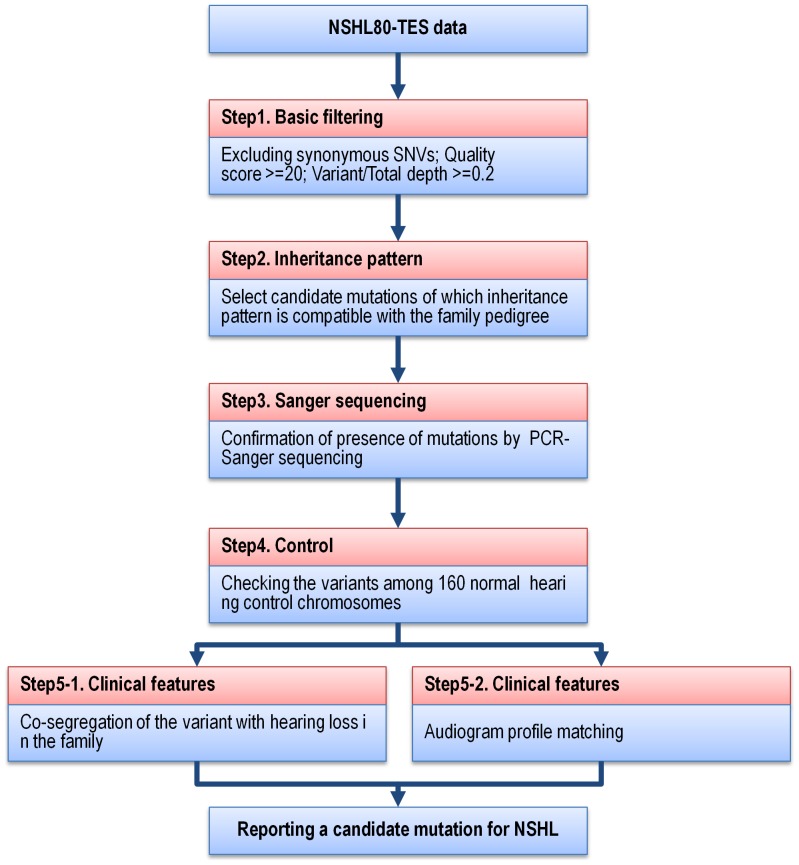
Analysis flow of NSHL-80 targeted resequencing on familial NSHL. Targeted resequencing data from 20 familial NSHL cases were filtered through five steps to select candidate SNVs in NSHL genes.

Next, we applied targeted resequencing for 20 probands of the remaining familial NSHL cases including one *GJB2* positive multiplex family (SH35) to screen all 80 reported NSHL-related genes (Fig. 1). We have captured 1254 exons of 80 genes (Table S2) spanning 480 kb in 20 probands from multiplex families for targeted exome sequencing. Mean read depth in 20 cases was 218.2±56.1 and 88.9±3.7% of bases was read in more than ×10 coverage (Table S3). About 90% exons in all patients were captured with ≥99% of bases at ≥10 of read depth. Missed or low-coverage exons were shared between samples, though different experimental procedures shared different uncaptured exons (Fig. S2). This ensures that most of captured exons were shared through samples, which does not disturb the following analysis of variant detection. The fraction of well-captured exons was much more than expectation by the model of independent uncaptured exons (Figs. S3 and S4).

**Figure 2:**
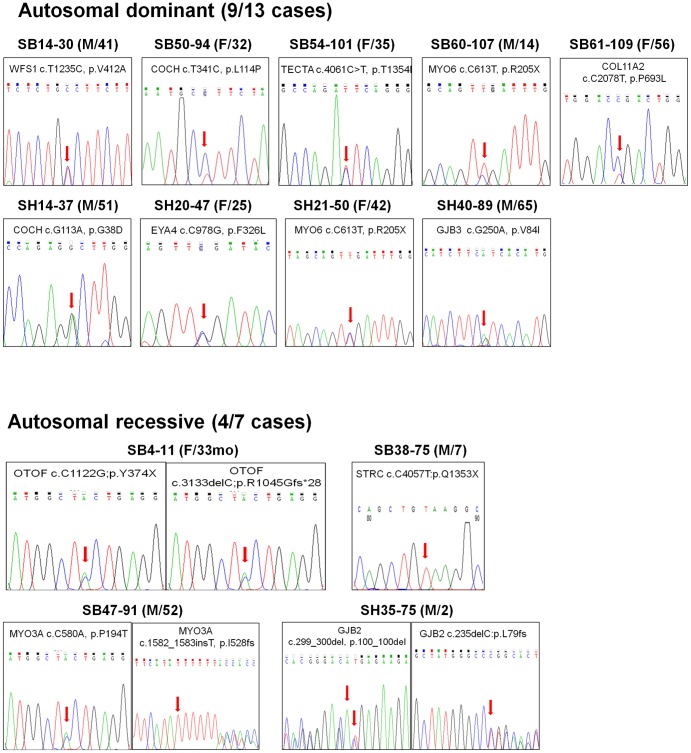
Validation of candidate mutations by PCR-Sanger sequencing. Candidate mutations in 9 autosomal dominant and 4 autosomal recessive NSHL families were shown in chromatogram of Sanger sequencing.

We selected rare single nucleotide variations (SNV) or indels following five steps of filtering to find candidate mutations related to hearing loss in each patient (Table 2). In a basic filtering step, variations with a quality score of less than 20 were discarded, and for heterozygous alleles, only the alleles with a ratio (coverage of variant over the total coverage) of 20% or more were included. The average number of variants was 4.8±0.42 per patient after basic filtering. As a second step, we checked inheritance pattern of multiplex family of each proband (Fig. 1), and excluded the variants which were not matched with the patient's inheritance pattern. According to the information on the inheritance, we could significantly reduce the average numbers of candidate mutations to 1.95±0.29 per patient (t-test p = 2.8×10^−6^). In three families, all the mutations were not matched with the inheritance pattern. In the third step, we validated 39 variants from the 18 probands by Sanger sequencing and confirmed 36 variants (92.3%) (Fig. 2). When we checked 80 normal hearing control subjects for the variants by Sanger sequencing, seven variants were also found in Korean population. As a final step for the filtering, we investigated segregation and/or phenotype matching to confirm the causality of the variant for deafness. Nineteen variants were examined in nine families by Sanger sequencing in all the family members to exclude 8 variants. We also examined patient's audiogram to match the candidate genes with the patient's phenotype and ruled out eleven variants, too. Especially, in cases where the segregation study could not be performed, we relied upon the audiogram configuration. Molecular genetic diagnosis was made in four subjects (SB61-109, SB55-102, SB50-94 and SB47-91) despite the lack of segregation study results, since their audiograms were well matched the previously reported characteristic audiogram configuration. Finally, we were able to find a most likely causative mutation in 13 out of 20 multiplex hearing loss families (Table 3).

**Table 2:** Number of candidate SNVs in 20 familial NSHL through five filtering steps.

Patient	1) basic filtering	2) inheritance pattern	3) Sanger sequencing	4) Control	5) Clinical feature		Final
					Segregation	Audiogram profile matching	
**ADNSHL**							
SB14-30	8	1	1	1	-	1	**1**
SB40-77	6	3	3	2	-	0	0
SB41-78	3	1	1	0	-	-	0
SB50-94	3	2	1	1	-	1	**1**
SB54-101	5	4	4	3	1	1	**1**
SB55-102	1	0	-	-	-	0	0
SB60-107	6	3	2	2	1	1	**1**
SB61-109	4	2	2	1	-	1	**1**
SH14-37	4	2	2	1	1	1	**1**
SH20-47	5	1	1	1	1	1	**1**
SH21-50	4	3	3	2	1	1	**1**
SH40-89	6	5	5	5	2	1	**1**
SH41-90	2	1	1	1	0	0	**0**
**ARNSHL**							
SB04-11	8	2	2	2	2	2	**2**
SB38-75	4	2	2	2	2	2	**2**
SB47-91	5	3	3	2	-	2	**2**
SH10-28	5	2	1	1	-	-	**0**
SH23-52	3	0	0	-	-	-	**0**
SH27-61	7	0	0	0	-	-	**0**
SH35-75	7	2	2	2	-	**?**	**2**

**Table 3:** List of final candidate SNVs in 13 familial NSHL.

Patient	Gene	Type	GeneBank No.	Chr	Exon	Nucleotide	Protein	Coverage of Ref	Coverage of Var	Quality score	1000g	dbsnp135
ADNSHL												
SB14-30	WFS1	Nonsynonymous	NM_006005	4	exon8	c.T1235C	p.V412A	118	131	99	0.0037	rs144951440
SB50-94	COCH	Nonsynonymous	NM_001135058	14	exon4	c.T341C	p.L114P	18	10	99	-	-
SB54-101	OTOR	stopgain	NM_020157	20	exon2	c.G223T	p.E75X	47	49	99	-	-
SB60-107	MYO6	stopgain	NM_004999	6	exon8	c.C613T	p.R205X	63	55	63,55	-	-
SB61-109	COL11A2	Nonsynonymous	NM_080680	6	exon30	c.C2336T	p.P779L	85	69	99	0.0005	rs150877886
SH14-37	COCH	Nonsynonymous	NM_001135058	14	exon3	c.G113A	p.G38D	79	78	99	-	-
SH20-47	EYA4	Nonsynonymous	NM_172103	6	exon11	c.C909G	p.F303L	69	52	99	-	-
SH21-50	MYO6	stopgain	NM_004999	6	exon8	c.C613T	p.R205X	41	51	99	-	-
SH40-89	GJB3	Nonsynonymous	NM_001005752	1	exon2	c.G250A	p.V84I	125	123	99	0.0018	rs145751680
ARNSHL												
SB04-11	OTOF	Frameshift deletion	NM_194322	2	exon24	c.3133delC	p.R1045Gfs*28	10	7	99	-	-
	OTOF	stopgain	NM_194322	2	exon8	c.C1122G	p.Y374X	75	52	99	-	-
SB38-75	STRC	stopgain	NM_153700	15	exon20	c.C4057T	p.Q1353X	0	39	81.2	-	rs2614824
SB47-91	MYO3A	Nonsynonymous	NM_017433	10	exon7	c.C580A	p.P194T	117	109	99	-	-
	MYO3A	Frameshift insertion	NM_017433	10	exon16	c.1582_1583insT	p.Y530Lfs*9	22	13	99	-	-
SH35-75	GJB2	Frameshift deletion	NM_004004	13	exon2	c.299_300del	p.H100Rfs*14	110	86	99	-	rs111033204
	GJB2	Frameshift deletion	NM_004004	13	exon2	c.235delC	p.L79Cfs*3	123	104	99	0.0023	rs80338943

**Figure 3:**
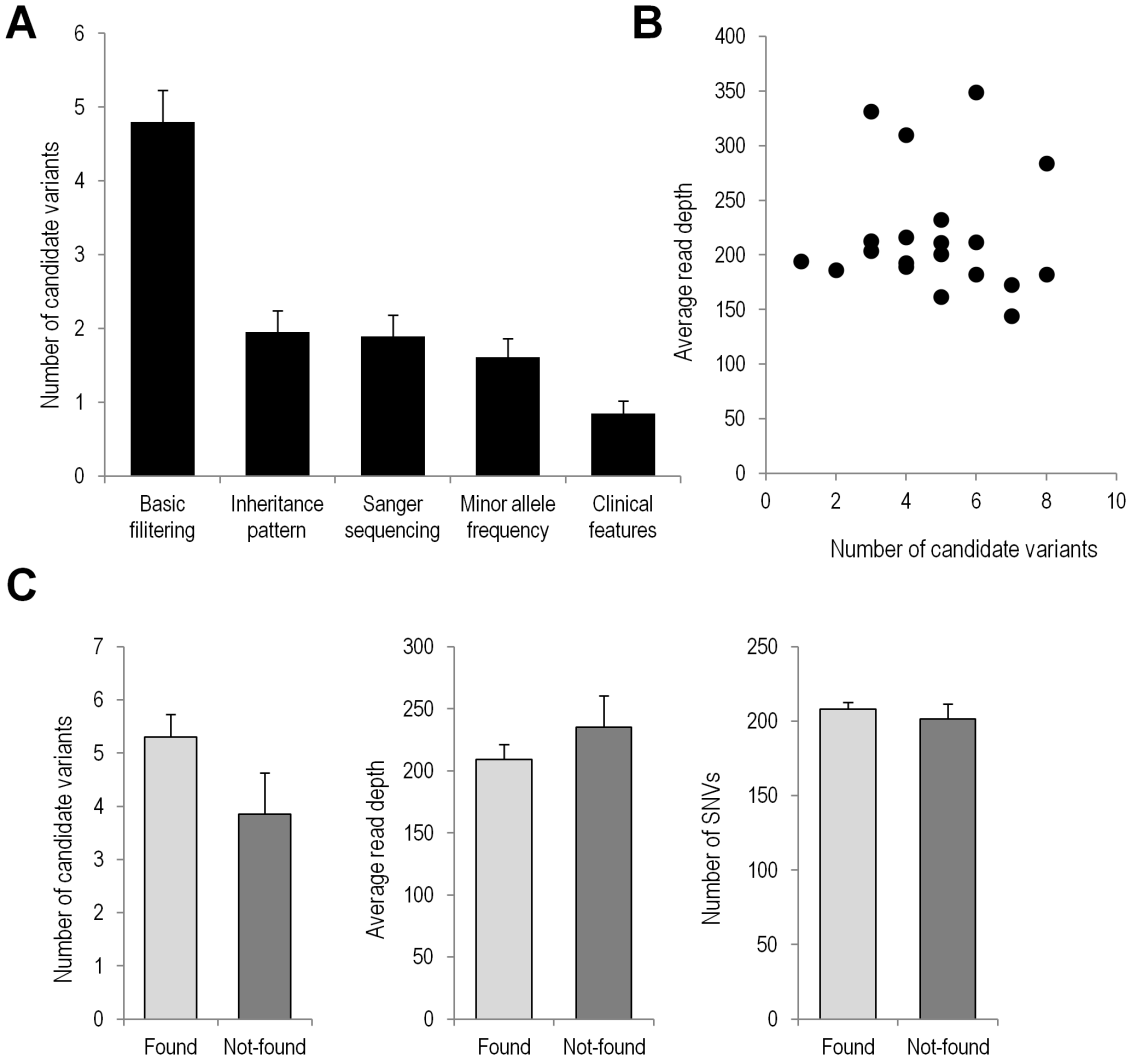
Interpretation of targeted resequencing in 20 probands. (A) An average number of candidate SNVs with standard errors were shown at five filtering steps. (B) The relationship between the numbers of candidate SNVs and read depth were plotted in 20 probands. (C) Candidate SNV-found patient group (Found) was compared with patient group without candidate SNV (Not-found) in the number of candidate variants, read depth and called SNVs.

Among 32 familial NSHL cases, we could detect mutations in 25 probands (79.1%) by Sanger and targeted exome sequencing in total. Breaking into the results depending upon the inheritance pattern, we were able to make a molecular genetic diagnosis from 9 (69.2%) of 13 autosomal dominant families on. seven genes such as *WFS1*, *COCH*, *EYA4*, *MYO6*, *GJB3*, *COL11A2* and *OTOR*. Molecular genetic diagnosis was possible in 9 (75.0%) of 12 recessive families. The four probably or possibly damaging mutations that we found were in *SLC26A4, GJB2, MYO3A, OTOF, and STRC*. We also found one case with *MRNR1* mutation with maternal inheritance, and five cases of *POU3F4* mutation with X-linked inheritance. However, we could not detect candidate mutations in seven probands, in which the number of variants from basic filtering was not correlated with read depth in 20 probands (Fig. 3A). The number of called variants, sequencing depth and mean coverage was not different from those with candidate mutations detected (Figs. 3B and 3C).

## Discussion

Genetic cause of sensorineural disorders such as mental retardation, retinitis pigmentosa, and congenital hearing loss is extraordinarily heterogeneous. It is hard to detect disease-causing mutation in each patient because we have to screen all the candidate genes. However, the genetic diagnosis by high-throughput sequence analysis helps clinicians and patients to delineate the characteristics of disease. Especially early intervention of hearing loss in children might provide better clinical outcomes in the linguistic development. In this study, we could enhance the efficiency to find genetic alterations in familial NSHL patients. A candidate gene approach using conventional PCR sequencing against the candidate genes related to a certain phenotypic marker can cover only 10–20% of familial NSHL cases. Methodologies that enable us to effectively screen these common mutations related to the certain phenotypic markers, such as multiplex SNaPshot minisequencing, have been introduced in our population [7]. Howerver, a substantial portion of NSHL cases without any phenotypic marker still remains unanswered in terms of the molecular genetic etiology.

**Figure 4:**
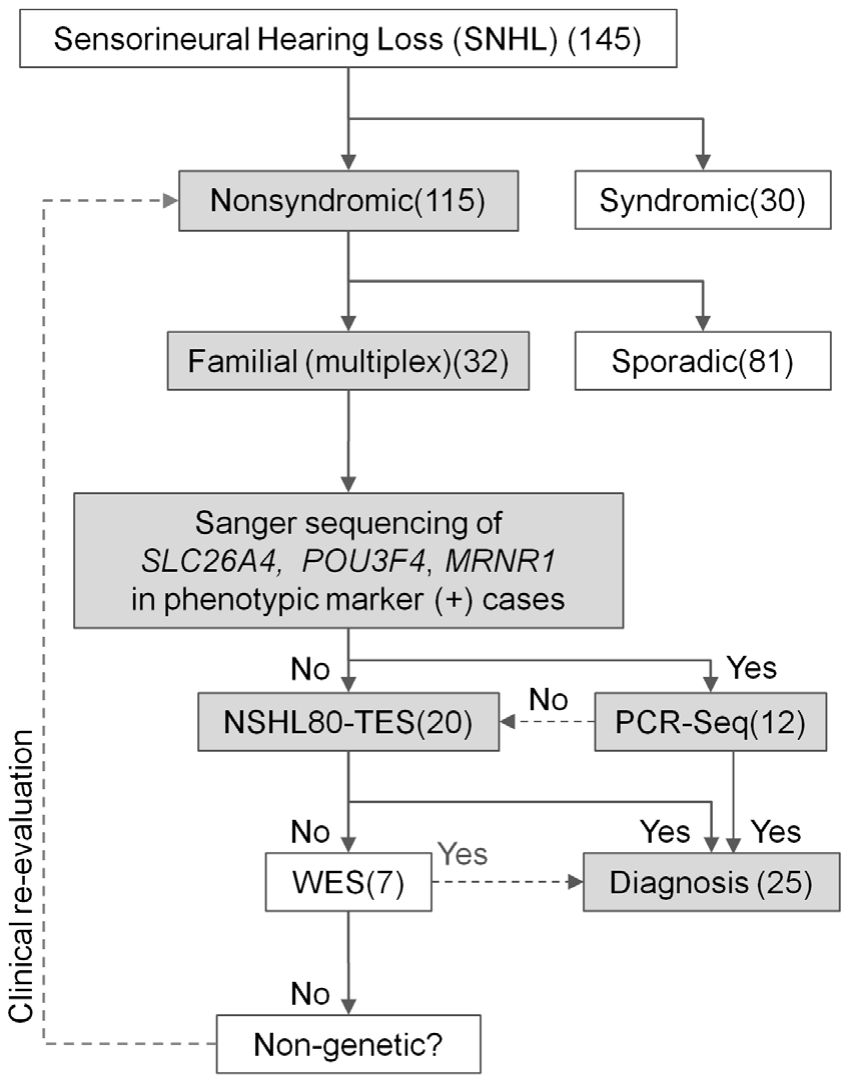
Proposed decision procedure for the genetic diagnosis of familial NSHL. We have recruited 145 sensorineural hearing loss patients, Among 115 NSHL cases, we started with 32 familial NSHL because we could check the inheritance patterns in the family. First, we excluded 12 cases with typical clinical features by PCR-Sanger sequencing. In the remaining 20 familial NSHL probands, we found candidate SNVs in 13 probands. In further study, we can find SNVs by whole exome sequencing (WES).

Therefore, we propose new diagnostic pipeline with high sensitivity to detect candidate mutations (Fig. 4).

Through the targeted resequencing of the 20 families, we found most likely responsible genes for nine out of thirteen AD families and four genes from seven AR families. Nevertheless, seven cases still need to find the final candidate mutation. The probands SB41-78 and SB55-102 have a relatively subtle phenotype considering their age (35 years old and 59 years old, respectively). Therefore, it is possible that their hearing loss is just a phenocopy. The proband SB40-77 showed characteristic mid frequency hearing loss', which rendered us to focus upon the candidate autosomal dominant genes such as *TECTA* or *COL11A2* that has been reported to cause ‘mid frequency hearing loss. However, we could not detect a candidate variant in those genes. Rather, we found a potentially pathogenic variant (c.G5054A:p.R1685Q) in the *MYH14* gene, a known deafness gene in DFNA4 locus from this proband. This variant has been detected neither in the 160 normal Korean control chromosomes nor in 1000 genomes. It was predicted to be ‘probably damaging’ by the Polyphen. In addition, the R1685 residue was conserved among many species including several mammalians, frog and zebrafish. However the audiogram pattern is not compatible with the previous reports [8], [9] and we were not able to check the segregation of the variant due to other family members’ reluctance to participate in this study (Table 2). Mutations in the different domains in the *MYH14* might lead to different audiogram configurations. Currently, we are thinking that this *MYH14* variant might account for the phenotype but did not count this as a causative mutation for this study. As for the family SH41, p.A2T variant of the *OTOR* gene (Accession No. AF233261) was detected after the basic filtering. This variant was not detected neither of normal 160 Korean chromosomes nor 1000 genomes. Robertson et al. (2000) proposed OTOR's possible role in human deafness based upon its preferential and abundant expression in the cochlea [10]. However, this variant did not co-segregate with hearing loss in one of the member in the family SH41.

Recently, narrow bony cochlear nerve canal (nBCNC) has been recognized and spotlighted as the most frequent inner ear anomaly [11]. Our group has postulated that the bilateral nBCNC may have a genetic etiology while the unilateral nBCNC is least likely to have a genetic contribution [12]. However, we were not able to find any candidate variant among the 80 deafness genes in the family SH27 where there was a sibling pair with bilateral nBCNC. The family SH23-52 segregates hearing loss presumably in an autosomal recessive manner, since the parents of three affected children showed perfect normal hearing. It is likely that the causative mutations for hearing loss in these families reside in genes other than 80 genes in this panel. We will further analyze the mutation in all exome of each family, because it may not be present in 80 candidate genes studied here.

Another reason for the detection failure may be due to the technical incompleteness. Coverage of targeted sequencing is not perfect to miss some exons, but usually considered good enough or not for the further analysis, especially in the experiments with many targets. This study also showed that 10% of exons were not properly captured. Capturing efficiency will be increased by new technologies for next generation sequencing. Recently, new enrichment technologies such as a semi-automated PCR amplification or a microdroplet PCR- based approach replacing the conventional hybridization-based enrichment technique have been successfully utilized in combination with next generation sequencing for genetic diagnosis of familial autosomal recessive deaf patients [13], [14]. However, the diagnostic yield in these studies was not greatly different from those in ours and previous studies utilizing the hybridization –based enrichment technique [15], [16], rendering us to believe that technical incompleteness in capturing cannot solely account for the detection failure.

We found most likely responsible genes for nine out of thirteen AD families. Among seven autosomal recessive NSHL cases, we could detect the mutations in four genes such as GJB2, MYO3A, STRC and OTOF in four cases. One of them was mutations in GJB2, which were used as a positive control because it is well known for causing severe prelingual hearing loss as for the patient SH35-75. The sequence analysis of candidate genes may be easier to use PCR method, but clinical decision for candidate gene sequencing may be more difficult for all the clinician. To this end, we propose simple single step sequence analysis using targeted exome sequencing.

## Supporting Information

Figure S1 Audiogram and pedigree for 12 familial NSHL used in PCR-Sanger sequencing.
(DOCX)

Figure S2 Audiogram and pedigree for 20 familial NSHL for targeted resequencing.
(DOCX)

Figure S3 Heatmap for percentage of bases ≥ depth 10, 50 or 100 within all target exons and samples. Most exons were uncaptured in common samples and samples were grouped by the common uncaptured exons.
(DOCX)

Figure S4 Barplot for comparison between experimental and expected distribution in number of exons that were commonly uncaptured within from 0 to 20 samples. Note that the exons in n = 0 were captured in all 20 samples and at n = 0, the experimental counts are greater than the expected ones due to common uncapured exons.
(DOCX)

Table S1 Primer sequences used for PCR-Sanger sequencing.
(DOCX)

Table S2 List of 80 genes related to NSHL for targeted resequencing.
(DOCX)

Table S3 Qualities of targeted resequencing in 20 familial NSHL.
(DOCX)
