# Supplementary figures and images for: Diagnostic Application of Targeted Resequencing for Familial Nonsyndromic Hearing Loss
Source: PLoS One. 2013 Aug 22;8(8):e68692. doi: 10.1371/journal.pone.0068692 (PMC3750053; doi:10.1371/journal.pone.0068692)

**
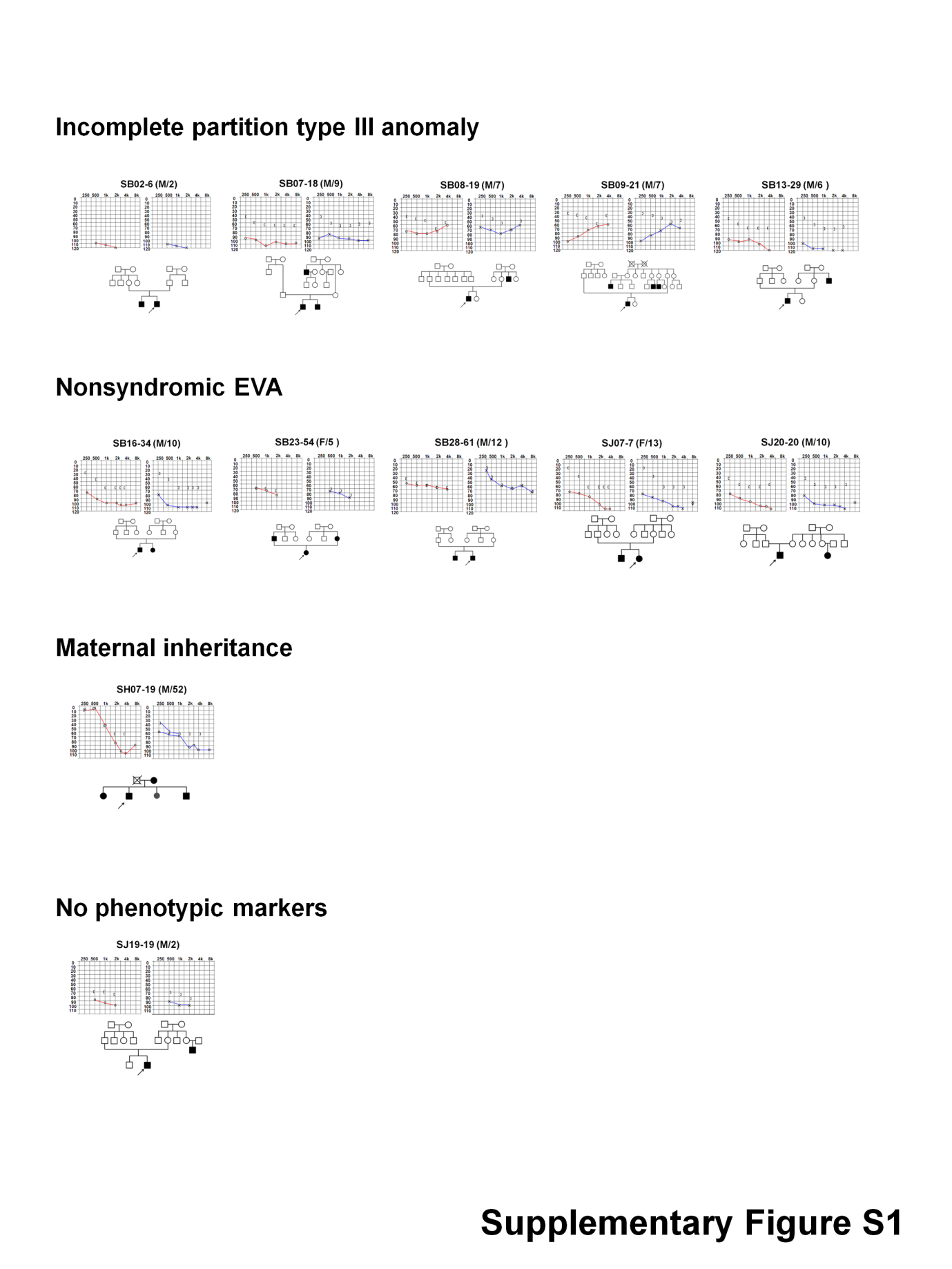
**

**Figure S1.** Audiogram and pedigree for 12 familial NSHL patients used in PCR-Sanger sequencing.

Supplement: Figure S1 — Audiogram and pedigree for 12 familial NSHL used in PCR-Sanger sequencing. (DOCX) [file pone.0068692.s001.docx]

**
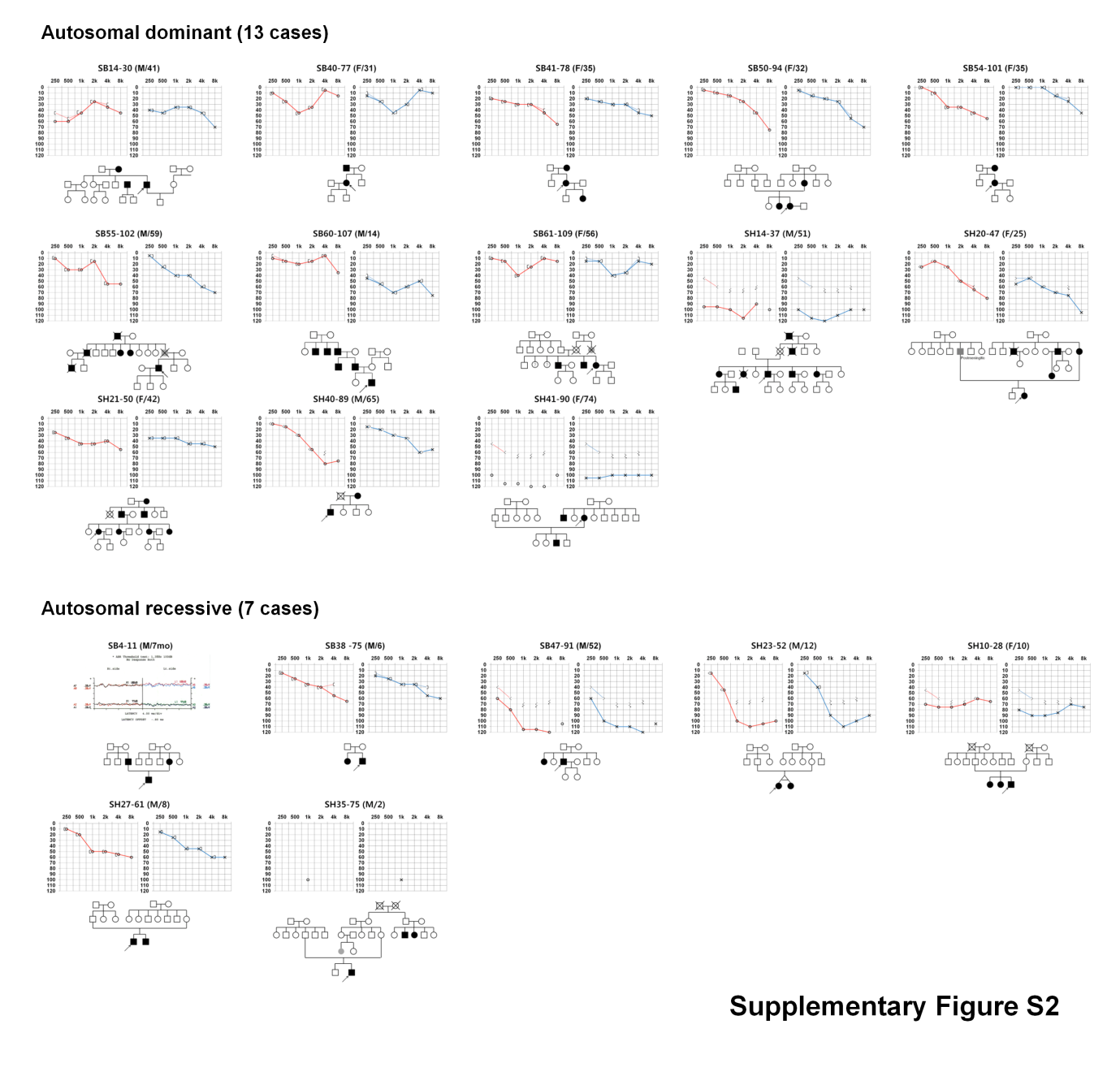
**

**Figure S2.** Audiogram and pedigree for 20 familial NSHL patients for targeted resequencing.

Supplement: Figure S2 — Audiogram and pedigree for 20 familial NSHL for targeted resequencing. (DOCX) [file pone.0068692.s002.docx]
