# Supplementary material for: Diagnostic Application of Targeted Resequencing for Familial Nonsyndromic Hearing Loss
Source: PLoS One. 2013 Aug 22;8(8):e68692. doi: 10.1371/journal.pone.0068692 (PMC3750053; doi:10.1371/journal.pone.0068692)
Supplement: Figure S3 — Heatmap for percentage of bases ≥ depth 10, 50 or 100 within all target exons and samples. Most exons were uncaptured in common samples and samples were grouped by the common uncaptured exons. (DOCX) [file pone.0068692.s003.docx]

**
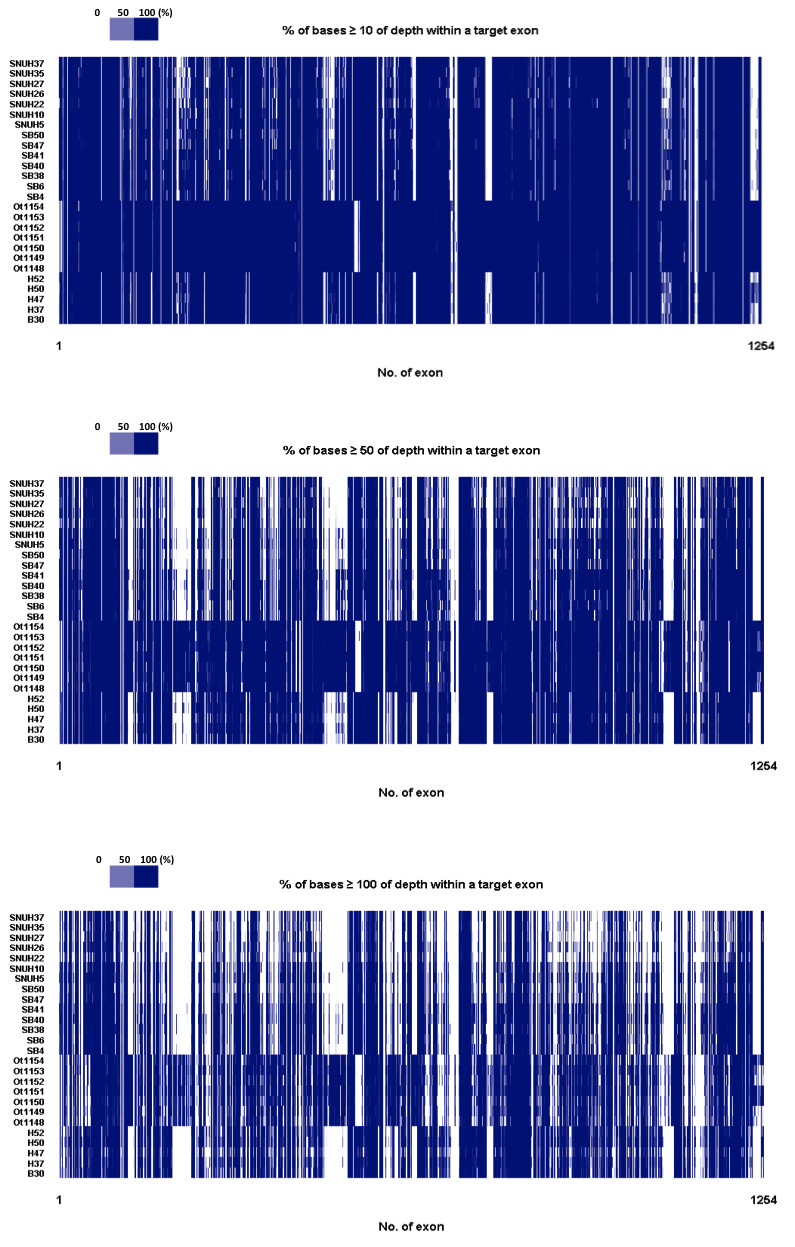
**

**Figure S3.** Heatmap for percentage of bases ≥ depth 10, 50 or 100 within all target exons and samples. Most exons were uncaptured in common samples, and samples were grouped by the common uncaptured exons.
