# Supplementary material for: Diagnostic Application of Targeted Resequencing for Familial Nonsyndromic Hearing Loss
Source: PLoS One. 2013 Aug 22;8(8):e68692. doi: 10.1371/journal.pone.0068692 (PMC3750053; doi:10.1371/journal.pone.0068692)
Supplement: Figure S4 — Barplot for comparison between experimental and expected distribution in number of exons that were commonly uncaptured within from 0 to 20 samples. Note that the exons in n = 0 were captured in all 20 samples and at n = 0, the experimental counts are greater than the expected ones due to common uncapured exons. (DOCX) [file pone.0068692.s004.docx]

**
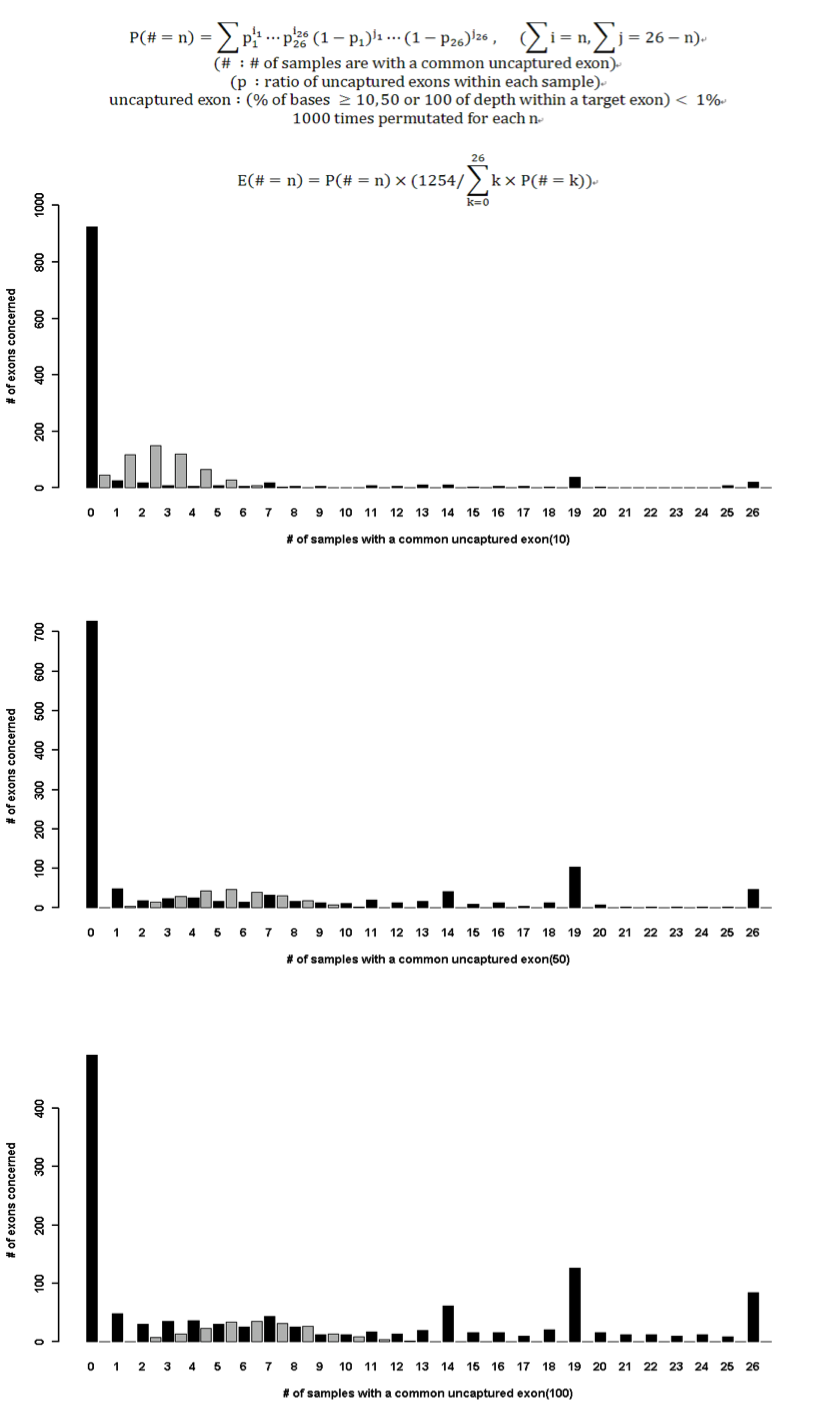
**

**Figure S4.** Barplot for comparison between experimental and expected distribution of number of exons that were commonly uncaptured from 0 to 20 samples. Note that the exons in n=0 were captured in all 20 samples, and at n=0, the experimental counts are greater than the expected ones due to common uncaptured exons.
