# Supplementary material for: Diagnostic Application of Targeted Resequencing for Familial Nonsyndromic Hearing Loss
Source: PLoS One. 2013 Aug 22;8(8):e68692. doi: 10.1371/journal.pone.0068692 (PMC3750053; doi:10.1371/journal.pone.0068692)
Supplement: Table S1 — Primer sequences used for PCR-Sanger sequencing. (DOCX) [file pone.0068692.s005.docx]

| **Table S1.** Primer sequences used for PCR-Sanger sequencing. | | | |  |  |
| --- | --- | --- | --- | --- | --- |
| **Gene/variant** | **Exon** | **Forward primer** | **Reverse primer** | | **Amplicon (bp)** |
| COCH | Exon 4 | GATGCCCTGAAAAAGTGTGG | TCACAGGTTTTTCCATCAAGG | | 289 |
|  | Exon 3 | AAAACAACCTTGTGGCTTGC | CCAGATGGGTAAAGCAGGAA | | 408 |
| COL11A2 | Exon 30 | CCCATCCTGACCCCAGTG | CACTGTTGCCCATTTCTCCT | | 249 |
| EYA4 | Exon 11 | GCCATCAGGAGGTTTCTATTGTAT | GTAGGCATACCTCCAGGTCACTAT | | 259 |
| GJB2 | Exon 2 | TCCTAGCTAGTGATTCCTGTGTTG | AGCCGTCGTACATGACATAGAAG | | 547 |
| GJB3 | Exon 2 | GATGAGCAGAAGGACTTTGACTG | AACTCAATGATGAGCTTGAAGATG | | 293 |
| MYO3A | Exon 7 | GCAATTGAAAGCTCTTTATATGAGT | AAAAGGAAAGTCAAACAGGATCAG | | 199 |
|  | Exon 16 | ATATCAGCACTCACAGTCGTTGTT | TAAAATTAAGGGAAAAGTGAAA | | 263 |
| MYO6 | Exon 8 | TATTTTGTAATGTTCCGTCATGCT | TCCTGCAACCATCTAAAGTAACAA | | 286 |
| OTOF | Exon 24 | TCAGTCCCTCCCATGCAG | TGTGCTGACCCCAAGACC | | 244 |
|  | Exon 8 | GGTGATCACACCTGTCCCTTA | CAACTCCCAGCCTCCAGTC | | 327 |
| OTOR | Exon 2 | GGGAATTATCAGTCACTCTGATTTT | TGAAGGTAGGTAATCAAGGAAAGG | | 314 |
| STRC | Exon 20 | CTCCAGTCTCAGGGGAAGTG | TCTGAAGTTCTCGAAGGTCCA | | 208 |
| WFS1 | Exon 8 | ATCGACTTCTTCGCCTTCTTC | CTTGAATTGGCCCTACCTGAAG | | 435 |
| MTRNR1 | mitochondria | TCTACCCCAGAAAACTACGATAGC | TGTTAAGCTACACTCTGGTTCGTC | | 248 |
