# Supplementary material for: Diagnostic Application of Targeted Resequencing for Familial Nonsyndromic Hearing Loss
Source: PLoS One. 2013 Aug 22;8(8):e68692. doi: 10.1371/journal.pone.0068692 (PMC3750053; doi:10.1371/journal.pone.0068692)
Supplement: Table S2 — List of 80 genes related to NSHL for targeted resequencing. (DOCX) [file pone.0068692.s006.docx]

| **Table S2.** List of 80 genes related to NSHL for targeted resequencing. | | | |
| --- | --- | --- | --- |
| **Symbol** | **Annotation** | **HGNC** | **OMIM** |
| ACTB | actin, beta | 132 | [607371] DYSTONIA, JUVENILE-ONSET |
| ACTG1 | actin, gamma 1 | 144 | [604717] DEAFNESS, AUTOSOMAL DOMINANT 20; DFNA20 |
| ATP6V1B1 | ATPase, H+ transporting, lysosomal 56/58kDa, V1 subunit B1 | 853 | [267300] RENAL TUBULAR ACIDOSIS, DISTAL, WITH PROGRESSIVE NERVE DEAFNESS |
| BCS1L | BCS1-like (S. cerevisiae) | 1020 | [603358] GRACILE SYNDROME [262000] BJORNSTAD SYNDROME; BJS [256000] LEIGH SYNDROME; LS [124000] MITOCHONDRIAL COMPLEX III DEFICIENCY |
| BSND | Bartter syndrome, infantile, with sensorineural deafness (Barttin) | 16512 | [602522] BARTTER SYNDROME, TYPE 4A |
| CATSPER2 | cation channel, sperm associated 2 | 18810 | [611102] DEAFNESS, SENSORINEURAL, AND MALE INFERTILITY |
| CCDC50 | coiled-coil domain containing 50 | 18111 | [607453] DEAFNESS, AUTOSOMAL DOMINANT 44; DFNA44 |
| CDH23 | cadherin-related 23 | 13733 | [601386] DEAFNESS, AUTOSOMAL RECESSIVE 12; DFNB12 [601067] USHER SYNDROME, TYPE ID; USH1D [276900] USHER SYNDROME, TYPE I; USH1 |
| CLDN14 | claudin 14 | 2035 | [614035] DEAFNESS, AUTOSOMAL RECESSIVE 29; DFNB29 |
| COCH | coagulation factor C homolog, cochlin (Limulus polyphemus) | 2180 | [601369] DEAFNESS, AUTOSOMAL DOMINANT 9; DFNA9 |
| COL11A2 | collagen, type XI, alpha 2 | 2187 | [614524] FIBROCHONDROGENESIS 2; FBCG2 [609706] DEAFNESS, AUTOSOMAL RECESSIVE 53; DFNB53 [601868] DEAFNESS, AUTOSOMAL DOMINANT 13; DFNA13 [277610] WEISSENBACHER-ZWEYMULLER SYNDROME; WZS [215150] OTOSPONDYLOMEGAEPIPHYSEAL DYSPLASIA; OSMED [184840] STICKLER SYNDROME, TYPE III; STL3 |
| COL9A3 | collagen, type IX, alpha 3 | 2219 | [603932] INTERVERTEBRAL DISC DISEASE; IDD [600969] EPIPHYSEAL DYSPLASIA, MULTIPLE, 3; EDM3 |
| CRYM | crystallin, mu | 2418 | #N/A |
| DFNA5 | deafness, autosomal dominant 5 | 2810 | [600994] DEAFNESS, AUTOSOMAL DOMINANT 5; DFNA5 |
| DFNB31 | deafness, autosomal recessive 31 | 16361 | [611383] USHER SYNDROME, TYPE IID; USH2D [607084] DEAFNESS, AUTOSOMAL RECESSIVE 31; DFNB31 |
| DFNB59 | deafness, autosomal recessive 59 | 29502 | [610220] DEAFNESS, AUTOSOMAL RECESSIVE 59; DFNB59 |
| DIAPH1 | diaphanous homolog 1 (Drosophila) | 2876 | [124900] DEAFNESS, AUTOSOMAL DOMINANT 1; DFNA1 |
| DSPP | dentin sialophosphoprotein | 3054 | [605594] DEAFNESS, AUTOSOMAL DOMINANT 39, WITH DENTINOGENESIS IMPERFECTA 1 [125500] DENTINOGENESIS IMPERFECTA, SHIELDS TYPE III [125490] DENTINOGENESIS IMPERFECTA 1; DGI1 [125420] DENTIN DYSPLASIA, TYPE II |
| ERCC2 | excision repair cross-complementing rodent repair deficiency, complementation group 2 | 3434 | [610756] CEREBROOCULOFACIOSKELETAL SYNDROME 2; COFS2 [601675] TRICHOTHIODYSTROPHY, PHOTOSENSITIVE; TTDP [278730] XERODERMA PIGMENTOSUM, COMPLEMENTATION GROUP D; XPD |
| ERCC3 | excision repair cross-complementing rodent repair deficiency, complementation group 3 (xeroderma pigmentosum group B complementing) | 3435 | [610651] XERODERMA PIGMENTOSUM, COMPLEMENTATION GROUP B; XPB [601675] TRICHOTHIODYSTROPHY, PHOTOSENSITIVE; TTDP |
| ESPN | espin | 13281 | [609006] DEAFNESS, AUTOSOMAL RECESSIVE 36, WITH OR WITHOUT VESTIBULAR INVOLVEMENT |
| ESRRB | estrogen-related receptor beta | 3473 | [608565] DEAFNESS, AUTOSOMAL RECESSIVE 35; DFNB35 |
| EYA4 | eyes absent homolog 4 (Drosophila) | 3522 | [605362] CARDIOMYOPATHY, DILATED, 1J; CMD1J [601316] DEAFNESS, AUTOSOMAL DOMINANT 10; DFNA10 |
| FGF3 | fibroblast growth factor 3 | 3681 | [610706] DEAFNESS, CONGENITAL, WITH INNER EAR AGENESIS, MICROTIA, AND MICRODONTIA |
| GATA3 | GATA binding protein 3 | 4172 | [146255] HYPOPARATHYROIDISM, SENSORINEURAL DEAFNESS, AND RENAL DISEASE; HDR |
| GJA1 | gap junction protein, alpha 1, 43kDa | 4274 | [600309] ATRIOVENTRICULAR SEPTAL DEFECT 3; AVSD3 [257850] OCULODENTODIGITAL DYSPLASIA, AUTOSOMAL RECESSIVE [241550] HYPOPLASTIC LEFT HEART SYNDROME 1; HLHS1 [234100] HALLERMANN-STREIFF SYNDROME; HSS [186100] SYNDACTYLY, TYPE III [164200] OCULODENTODIGITAL DYSPLASIA; ODDD |
| GJB1 | gap junction protein, beta 1, 32kDa | 4283 | [302800] CHARCOT-MARIE-TOOTH DISEASE, X-LINKED DOMINANT, 1; CMTX1 [145900] HYPERTROPHIC NEUROPATHY OF DEJERINE-SOTTAS |
| GJB2 | gap junction protein, beta 2, 26kDa | 4284 | [602540] ICHTHYOSIS, HYSTRIX-LIKE, WITH DEAFNESS [601544] DEAFNESS, AUTOSOMAL DOMINANT 3A; DFNA3A [220290] DEAFNESS, AUTOSOMAL RECESSIVE 1A; DFNB1A [149200] KNUCKLE PADS, LEUKONYCHIA, AND SENSORINEURAL DEAFNESS [148350] KERATODERMA, PALMOPLANTAR, WITH DEAFNESS [148210] KERATITIS-ICHTHYOSIS-DEAFNESS SYNDROME, AUTOSOMAL DOMINANT [124500] DEAFNESS, CONGENITAL, WITH KERATOPACHYDERMIA AND CONSTRICTIONS OF |
| GJB3 | gap junction protein, beta 3, 31kDa | 4285 | [612644] DEAFNESS, AUTOSOMAL DOMINANT 2B; DFNA2B [603324] GAP JUNCTION PROTEIN, BETA-3; GJB3 [600101] DEAFNESS, AUTOSOMAL DOMINANT 2A; DFNA2A [220290] DEAFNESS, AUTOSOMAL RECESSIVE 1A; DFNB1A [133200] ERYTHROKERATODERMIA VARIABILIS ET PROGRESSIVA; EKVP |
| GJB4 | gap junction protein, beta 4, 30.3kDa | 4286 | [133200] ERYTHROKERATODERMIA VARIABILIS ET PROGRESSIVA; EKVP |
| GJB6 | gap junction protein, beta 6, 30kDa | 4288 | [612645] DEAFNESS, AUTOSOMAL RECESSIVE 1B; DFNB1B [612643] DEAFNESS, AUTOSOMAL DOMINANT 3B; DFNA3B [601544] DEAFNESS, AUTOSOMAL DOMINANT 3A; DFNA3A [220290] DEAFNESS, AUTOSOMAL RECESSIVE 1A; DFNB1A [129500] ECTODERMAL DYSPLASIA, HIDROTIC, AUTOSOMAL DOMINANT |
| GRHL2 | grainyhead-like 2 (Drosophila) | 2799 | [608641] DEAFNESS, AUTOSOMAL DOMINANT 28; DFNA28 |
| GSTP1 | glutathione S-transferase pi 1 | 4638 | #N/A |
| JAG1 | jagged 1 | 6188 | [601920] JAGGED 1; JAG1 [187500] TETRALOGY OF FALLOT; TOF [118450] ALAGILLE SYNDROME 1; ALGS1 |
| KCNE1 | potassium voltage-gated channel, Isk-related family, member 1 | 6240 | [613695] LONG QT SYNDROME 5; LQT5 [612347] JERVELL AND LANGE-NIELSEN SYNDROME 2; JLNS2 [220400] JERVELL AND LANGE-NIELSEN SYNDROME 1; JLNS1 |
| KCNJ10 | potassium inwardly-rectifying channel, subfamily J, member 10 | 6256 | [612780] SEIZURES, SENSORINEURAL DEAFNESS, ATAXIA, MENTAL RETARDATION, AND [600791] ENLARGED VESTIBULAR AQUEDUCT; EVA |
| KCNQ4 | potassium voltage-gated channel, KQT-like subfamily, member 4 | 6298 | [600101] DEAFNESS, AUTOSOMAL DOMINANT 2A; DFNA2A |
| LHFPL5 | lipoma HMGIC fusion partner-like 5 | 21253 | [610265] DEAFNESS, AUTOSOMAL RECESSIVE 67; DFNB67 |
| LHX3 | LIM homeobox 3 | 6595 | [262600] PITUITARY HORMONE DEFICIENCY, COMBINED, 2; CPHD2 [221750] PITUITARY HORMONE DEFICIENCY, COMBINED, 3; CPHD3 |
| LRTOMT | leucine rich transmembrane and 0-methyltransferase domain containing | 25033 | [611451] DEAFNESS, AUTOSOMAL RECESSIVE 63; DFNB63 |
| MARVELD2 | MARVEL domain containing 2 | 26401 | [610153] DEAFNESS, AUTOSOMAL RECESSIVE 49; DFNB49 |
| MTAP | methylthioadenosine phosphorylase | 7413 | #N/A |
| MYH14 | myosin, heavy chain 14, non-muscle | 23212 | [614369] PERIPHERAL NEUROPATHY, MYOPATHY, HOARSENESS, AND HEARING LOSS; PNMHH [600652] DEAFNESS, AUTOSOMAL DOMINANT 4; DFNA4 |
| MYH9 | myosin, heavy chain 9, non-muscle | 7579 | [605249] SEBASTIAN SYNDROME; SBS [603622] DEAFNESS, AUTOSOMAL DOMINANT 17; DFNA17 [600208] MACROTHROMBOCYTOPENIA AND PROGRESSIVE SENSORINEURAL DEAFNESS [155100] MAY-HEGGLIN ANOMALY; MHA [153650] EPSTEIN SYNDROME, [153640] FECHTNER SYNDROME; FTNS |
| MYO15A | myosin XVA | 7594 | [600316] DEAFNESS, AUTOSOMAL RECESSIVE 3; DFNB3 |
| MYO1A | myosin IA | 7595 | [607841] DEAFNESS, AUTOSOMAL DOMINANT 48; DFNA48 |
| MYO1C | myosin IC | 7597 | #N/A |
| MYO1F | myosin IF | 7600 | #N/A |
| MYO3A | myosin IIIA | 7601 | [607101] DEAFNESS, AUTOSOMAL RECESSIVE 30; DFNB30 |
| MYO6 | myosin VI | 7605 | [607821] DEAFNESS, AUTOSOMAL RECESSIVE 37; DFNB37 [606346] DEAFNESS, AUTOSOMAL DOMINANT 22; DFNA22 |
| MYO7A | myosin VIIA | 7606 | [601317] DEAFNESS, AUTOSOMAL DOMINANT 11; DFNA11 [600060] DEAFNESS, AUTOSOMAL RECESSIVE 2; DFNB2 [276900] USHER SYNDROME, TYPE I; USH1 |
| NR2F1 | nuclear receptor subfamily 2, group F, member 1 | 7975 | #N/A |
| OTOA | otoancorin | 16378 | [607039] DEAFNESS, AUTOSOMAL RECESSIVE 22; DFNB22 |
| OTOF | otoferlin | 8515 | [601071] DEAFNESS, AUTOSOMAL RECESSIVE 9; DFNB9 |
| OTOR | otoraplin | 8517 | #N/A |
| PAX3 | paired box 3 | 8617 | [268220] RHABDOMYOSARCOMA 2; RMS2 [193500] WAARDENBURG SYNDROME, TYPE 1; WS1 [148820] WAARDENBURG SYNDROME, TYPE 3; WS3 [122880] CRANIOFACIAL-DEAFNESS-HAND SYNDROME; CDHS |
| PCDH15 | protocadherin-related 15 | 14674 | [609533] DEAFNESS, AUTOSOMAL RECESSIVE 23; DFNB23 [602083] USHER SYNDROME, TYPE IF; USH1F [601067] USHER SYNDROME, TYPE ID; USH1D [276900] USHER SYNDROME, TYPE I; USH1 |
| PDZD7 | PDZ domain containing 7 | 26257 | [605472] USHER SYNDROME, TYPE IIC; USH2C [276901] USHER SYNDROME, TYPE IIA; USH2A |
| PMP22 | peripheral myelin protein 22 | 9118 | [180800] ROUSSY-LEVY HEREDITARY AREFLEXIC DYSTASIA [162500] NEUROPATHY, HEREDITARY, WITH LIABILITY TO PRESSURE PALSIES; HNPP [145900] HYPERTROPHIC NEUROPATHY OF DEJERINE-SOTTAS [139393] GUILLAIN-BARRE SYNDROME, FAMILIAL; GBS [118300] CHARCOT-MARIE-TOOTH DISEASE AND DEAFNESS [118220] CHARCOT-MARIE-TOOTH DISEASE, DEMYELINATING, TYPE 1A; CMT1A |
| POU4F3 | POU class 4 homeobox 3 | 9220 | [602459] DEAFNESS, AUTOSOMAL DOMINANT 15; DFNA15 |
| RDX | radixin | 9944 | [611022] DEAFNESS, AUTOSOMAL RECESSIVE 24; DFNB24 |
| SLC17A8 | solute carrier family 17 (sodium-dependent inorganic phosphate cotransporter), member 8 | 20151 | [605583] DEAFNESS, AUTOSOMAL DOMINANT 25; DFNA25 |
| SLC26A4 | solute carrier family 26, member 4 | 8818 | [600791] ENLARGED VESTIBULAR AQUEDUCT; EVA [274600] PENDRED SYNDROME; PDS |
| SLC26A5 | solute carrier family 26, member 5 (prestin) | 9359 | [613865] DEAFNESS, AUTOSOMAL RECESSIVE 61; DFNB61 |
| SLC4A11 | solute carrier family 4, sodium borate transporter, member 11 | 16438 | [613268] CORNEAL DYSTROPHY, FUCHS ENDOTHELIAL, 4; FECD4 [217700] CORNEAL ENDOTHELIAL DYSTROPHY 2, AUTOSOMAL RECESSIVE; CHED2 [217400] CORNEAL DYSTROPHY AND PERCEPTIVE DEAFNESS |
| SOX2 | SRY (sex determining region Y)-box 2 | 11195 | [206900] MICROPHTHALMIA, SYNDROMIC 3; MCOPS3 |
| SPINK5 | serine peptidase inhibitor, Kazal type 5 | 15464 | [256500] NETHERTON SYNDROME; NETH [147050] IgE RESPONSIVENESS, ATOPIC; IGER |
| STRC | stereocilin | 16035 | [611102] DEAFNESS, SENSORINEURAL, AND MALE INFERTILITY [603720] DEAFNESS, AUTOSOMAL RECESSIVE 16; DFNB16 |
| TBL1X | transducin (beta)-like 1X-linked | 11585 | #N/A |
| TCF21 | transcription factor 21 | 11632 | #N/A |
| TECTA | tectorin alpha | 11720 | [603629] DEAFNESS, AUTOSOMAL RECESSIVE 21; DFNB21 [601543] DEAFNESS, AUTOSOMAL DOMINANT 12; DFNA12 |
| TIMM8A | translocase of inner mitochondrial membrane 8 homolog A (yeast) | 11817 | [311150] OPTICOACOUSTIC NERVE ATROPHY WITH DEMENTIA [304700] MOHR-TRANEBJAERG SYNDROME; MTS |
| TMC1 | transmembrane channel-like 1 | 16513 | [606705] DEAFNESS, AUTOSOMAL DOMINANT 36; DFNA36 [600974] DEAFNESS, AUTOSOMAL RECESSIVE 7; DFNB7 |
| TMIE | transmembrane inner ear | 30800 | [600971] DEAFNESS, AUTOSOMAL RECESSIVE 6; DFNB6 |
| TMPRSS3 | transmembrane protease, serine 3 | 11877 | [605316] DEAFNESS, AUTOSOMAL RECESSIVE 10; DFNB10 [601072] DEAFNESS, CHILDHOOD-ONSET NEUROSENSORY, AUTOSOMAL RECESSIVE 8; DFNB8 |
| TMPRSS5 | transmembrane protease, serine 5 | 14908 | #N/A |
| TRIOBP | TRIO and F-actin binding protein | 17009 | [609823] DEAFNESS, AUTOSOMAL RECESSIVE 28; DFNB28 |
| USH1C | Usher syndrome 1C (autosomal recessive, severe) | 12597 | [602092] DEAFNESS, AUTOSOMAL RECESSIVE 18; DFNB18 [276904] USHER SYNDROME, TYPE IC; USH1C [276900] USHER SYNDROME, TYPE I; USH1 |
| WFS1 | Wolfram syndrome 1 (wolframin) | 12762 | [614296] WOLFRAM-LIKE SYNDROME, AUTOSOMAL DOMINANT; WFSL [600965] DEAFNESS, AUTOSOMAL DOMINANT 6; DFNA6 [222300] WOLFRAM SYNDROME 1; WFS1 [125853] DIABETES MELLITUS, NONINSULIN-DEPENDENT; NIDDM |
