# Supplementary material for: Diagnostic Application of Targeted Resequencing for Familial Nonsyndromic Hearing Loss
Source: PLoS One. 2013 Aug 22;8(8):e68692. doi: 10.1371/journal.pone.0068692 (PMC3750053; doi:10.1371/journal.pone.0068692)
Supplement: Table S3 — Qualities of targeted resequencing in 20 familial NSHL. (DOCX) [file pone.0068692.s007.docx]

| **Table S3.** Qualities of targeted resequencing in 20 familial NSHL. | | | | | | | | |  | |  | | | |  |
| --- | --- | --- | --- | --- | --- | --- | --- | --- | --- | --- | --- | --- | --- | --- | --- |
| **Sample** | **Depth** | | | | | | |  | | **SNP** | | | | | **Indel** |
|  | **Mean** | **Q3** | **Median** | **Q1** | **%>=10** | **%>=50** | **%>=100** |  | | **Total** | | **synon** | **nonsyn** | **stopgain** | **Total** |
| SB14-30 | 182.0 | 253 | 154 | 62 | 88.2 | 77.4 | 65.4 |  | | 176 | | 100 | 75 | 1 | 9 |
| SB38-75 | 309.6 | 478 | 228 | 60 | 86.3 | 77.0 | 67.9 |  | | 204 | | 118 | 85 | 1 | 14 |
| SB40-77 | 348.8 | 500 | 266 | 65 | 87.3 | 77.8 | 70.0 |  | | 181 | | 94 | 87 | 0 | 17 |
| SB4-11 | 283.6 | 434 | 204 | 52 | 86.1 | 75.5 | 66.5 |  | | 190 | | 101 | 87 | 2 | 16 |
| SB41-78 | 331.2 | 500 | 247 | 63 | 86.6 | 77.2 | 69.0 |  | | 189 | | 108 | 79 | 2 | 12 |
| SB47-91 | 232.2 | 342 | 163 | 43 | 84.7 | 72.7 | 62.4 |  | | 203 | | 116 | 86 | 1 | 18 |
| SB50-94 | 212.6 | 313 | 147 | 34 | 84.5 | 71.0 | 59.6 |  | | 186 | | 99 | 87 | 0 | 19 |
| SB54-101 | 200.7 | 258 | 171 | 102 | 93.4 | 88.2 | 75.5 |  | | 214 | | 136 | 77 | 1 | 7 |
| SB55-102 | 194.1 | 250 | 163 | 97 | 93.3 | 88.0 | 74.0 |  | | 156 | | 87 | 69 | 0 | 10 |
| SB60-107 | 211.6 | 272 | 177 | 105 | 93.5 | 88.8 | 76.5 |  | | 213 | | 123 | 89 | 1 | 12 |
| SB61-109 | 189.2 | 242 | 161 | 96 | 93.3 | 87.7 | 73.3 |  | | 232 | | 140 | 92 | 0 | 15 |
| SH10-28 | 211.1 | 319 | 148 | 37 | 85.0 | 71.2 | 60.2 |  | | 198 | | 107 | 91 | 0 | 12 |
| SH14-37 | 192.7 | 268 | 164 | 67 | 88.0 | 78.4 | 67.1 |  | | 212 | | 128 | 84 | 0 | 12 |
| SH20-47 | 161.6 | 223 | 136 | 55 | 88.2 | 76.1 | 62.1 |  | | 224 | | 133 | 91 | 0 | 11 |
| SH21-50 | 216.1 | 303 | 186 | 75 | 89.0 | 79.5 | 70.5 |  | | 195 | | 105 | 88 | 2 | 11 |
| SH23-52 | 203.3 | 285 | 173 | 69 | 88.2 | 78.7 | 68.4 |  | | 227 | | 130 | 97 | 0 | 11 |
| SH27-61 | 172.4 | 222 | 144 | 85 | 93.2 | 86.9 | 68.7 |  | | 222 | | 126 | 96 | 0 | 18 |
| SH35-75 | 144.1 | 209 | 95 | 24 | 82.2 | 64.7 | 48.6 |  | | 221 | | 129 | 92 | 0 | 18 |
| SH40-89 | 181.9 | 235 | 150 | 90 | 93.2 | 87.4 | 70.7 |  | | 235 | | 143 | 92 | 0 | 14 |
| SH41-90 | 185.9 | 239 | 156 | 92 | 93.1 | 87.3 | 71.9 |  | | 237 | | 141 | 96 | 0 | 13 |
| Average | 218.2 | 307.3 | 171.7 | 68.7 | 88.9 | 79.6 | 67.4 |  | | 205.8 | | 118.2 | 87.0 | 0.6 | 13.5 |
| SD | 56.1 | 94.9 | 39.4 | 23.8 | 3.7 | 7 | 6.5 |  | | 21.8 | | 17.1 | 7.3 | 0.8 | 3.4 |
